# Three-Dimensional Imaging of the Developing Vasculature within Stem Cell-Seeded Scaffolds Cultured *in ovo*

**DOI:** 10.3389/fphys.2016.00146

**Published:** 2016-04-21

**Authors:** Anna Woloszyk, Davide Liccardo, Thimios A. Mitsiadis

**Affiliations:** ^1^Orofacial Development and Regeneration, Centre for Dental Medicine, Institute of Oral Biology, University of ZurichZurich, Switzerland; ^2^Section of Biotechnology and Medical Histology and Embryology, Department of Experimental Medicine, Second University of NaplesNaples, Italy

**Keywords:** chorioallantoic membrane (CAM), vascularization, stem cells, biomaterials, 3D imaging, MicroFil®, microcomputed tomography, regenerative medicine

## Abstract

Successful tissue engineering requires functional vascularization of the three-dimensional constructs with the aim to serve as implants for tissue replacement and regeneration. The survival of the implant is only possible if the supply of oxygen and nutrients by developing capillaries from the host is established. The chorioallantoic membrane (CAM) assay is a valuable tool to study the ingrowth and distribution of vessels into scaffolds composed by appropriate biomaterials and stem cell populations that are used in cell-based regenerative approaches. The developing vasculature of chicken embryos within cell-seeded scaffolds can be visualized with microcomputed tomography after intravenous injection of MicroFil®, which is a radiopaque contrast agent. Here, we provide a step-by-step protocol for the seeding of stem cells into silk fibroin scaffolds, the CAM culture conditions, the procedure of MicroFil® perfusion, and finally the microcomputed tomography scanning. Three-dimensional imaging of the vascularized tissue engineered constructs provides an important analytical tool for studying the potential of cell seeded scaffolds to attract vessels and form vascular networks, as well as for analyzing the number, density, length, branching, and diameter of vessels. This *in ovo* method can greatly help to screen implants that will be used for tissue regeneration purposes before their *in vivo* testing, thereby reducing the amount of animals needed for pre-clinical studies.

## Introduction

The formation of new networks of blood vessels (i.e., angiogenesis) is essential in embryonic development, tissue homeostasis, pathology, and regeneration. Hematopoietic cells circulating within the vasculature supply the surrounding tissues with oxygen and nutrients, transport hormones, remove waste products, and CO_2_, and protect the body from infections (Carmeliet and Jain, 2011). Successful engineering of tissue substitutes and transplantable organs depends on their rapid and adequate angiogenesis, since the appropriate supply of oxygen and nutrients is crucial for their long-term survival (Mitsiadis and Harada, 2015; Mitsiadis et al., 2015). A dense network of capillaries is formed in most tissues of the body that regulates the diffusion limit of oxygen (Carmeliet and Jain, 2000; Novosel et al., 2011). Spontaneous vascular ingrowth from the host tissue to the transplanted devices (e.g., empty biodegradable implants) is very slow and limited to several tenths of micrometers per day (Rouwkema et al., 2008). Therefore, the improvement and acceleration of implant vascularization remains a key challenge in regenerative medicine.

Classical *in vivo* angiogenesis assays rely on small (e.g., mice, rats) and large (e.g., sheep, goats) animal models, where engineered constructs are most commonly implanted subcutaneously at their dorsal part (Staton et al., 2009; Jungraithmayr et al., 2013; Wang et al., 2015). However, these surgical procedures are time-consuming and often associated with high costs and repetitive animal sacrifice. The chorioallantoic membrane (CAM) is highly vascularized and serves as a transient gas exchange surface during the incubation period, similar to the lung (Romanoff, 1960). Originally, the CAM assay was used in order to study the developmental potential of embryonic tissue grafts (Rücker et al., 2006). As chicken embryos become immunocompetent only by day 18 of their development (Janković et al., 1975), various biological processes where vascularization plays a role can be studied without inducing an immune response, including insufficient vascularization (e.g., ischemic disorders) or excessive vessel formation (e.g., cancer), with the goal to either enhance or decrease vessel growth, respectively (Ribatti, 2014).

To visualize the three-dimensional (3D) development of the vasculature within implants cultured on the CAM, perfusion of the chicken embryos was performed with the radiopaque contrast agent MicroFil® followed by microcomputed tomography (microCT) of the implants. We demonstrate that this technique, which is commonly used in *in vivo* experiments performed in rodents (Bolland et al., 2008; Schmidt et al., 2009; Stoppato et al., 2013), can also successfully be applied in the CAM assay.

## Materials and methods

### Seeding of human dental pulp stem cells

- Defrost cryopreserved human dental pulp stem cells and culture in Dulbecco's Modified Eagle Medium: Nutrient Mixture F-12 (DMEM/F12) + 10% fetal bovine serum + 1% penicillin/streptamycin + 0.5 μg/mL fungizone in a humidified incubator at 37°C and 5% CO_2_. Change the medium every 3–4 days.
- At 80–90% confluency, trypsinize the cells, use a 70 μm strainer to obtain a single-cell suspension, centrifuge (1500 rpm, 5 min, RT), and resuspend the cell pellet at a concentration of 1 × 10^6^ cells/50 μL.
- Place the sterile cylindrical scaffold (silk fibroin, 5 mm diameter, 3 mm height) on a sterile filter paper to absorb any residual liquid and move the scaffold into the well of a 96-well-plate.
- Seed the scaffold by pipetting 50 μL of the cell suspension on top of the scaffold, turn the scaffold, take up the cell suspension from the bottom of the well, and release it again on top of the scaffold.
- For cell attachment, place the cell-seeded scaffolds in a humidified incubator for 1 h at 37°C and 5% CO_2_. Wash the scaffold before placing it in a clean well of a 24-well-plate and adding 1 mL of medium. Culture for desired period of time and change medium every 3–4 days.

The procedure for anonymized cell collection was approved by the Kantonale Ethikkommission of Zurich and performed with written patients' consent.

### Preparation of the chicken eggs for the CAM assay and placing of the cell-seeded samples

- Pre-incubate fertilized Lohman white LSL (Lohman Selected Leghorn) chicken eggs in an egg incubator for 3 days at 38°C at a rotation speed of 360°/4 h.
- On embryonic day 3 (ED 3), stop the rotation in the morning and let the eggs rest in this position for 3 h to ensure that the embryo is on the top.
- Mark the top of the egg with a pencil and carefully wipe the egg-shell with 70% ethanol without turning it.
- Inside a clean bench, place the egg in a 60 mm Petri dish on top of a piece of tape to stabilize the egg.
- Make a small hole in the shell with the tip of sterile pointy scissors and remove 4 mL of albumen using a syringe and a needle to lower the developing embryo.
- Put Scotch^TM^ tape on the area where you want to make the window.
- Make another small hole, insert the scissors carefully and start cutting an oval hole while turning the egg with the other hand.
- Remove the shell and cover the opening with a second 60 mm Petri dish, which needs to be fixed to the bottom lid with tape, and incubate in a humidified incubator at 37°C and 0% CO_2_.
- On ED 7, the cell-seeded scaffolds can be placed on the vascularized CAM. Remove the Petri dish lid, place a silicone ring (taken from sterile cryovials) on the CAM to ensure a flat surface, and position the sample in the middle of this ring.
- Incubate for 7 days until ED 14 in a humidified incubator at 37°C and 0% CO_2_.

### Microfil® perfusion and microct imaging

**Figure 1:**
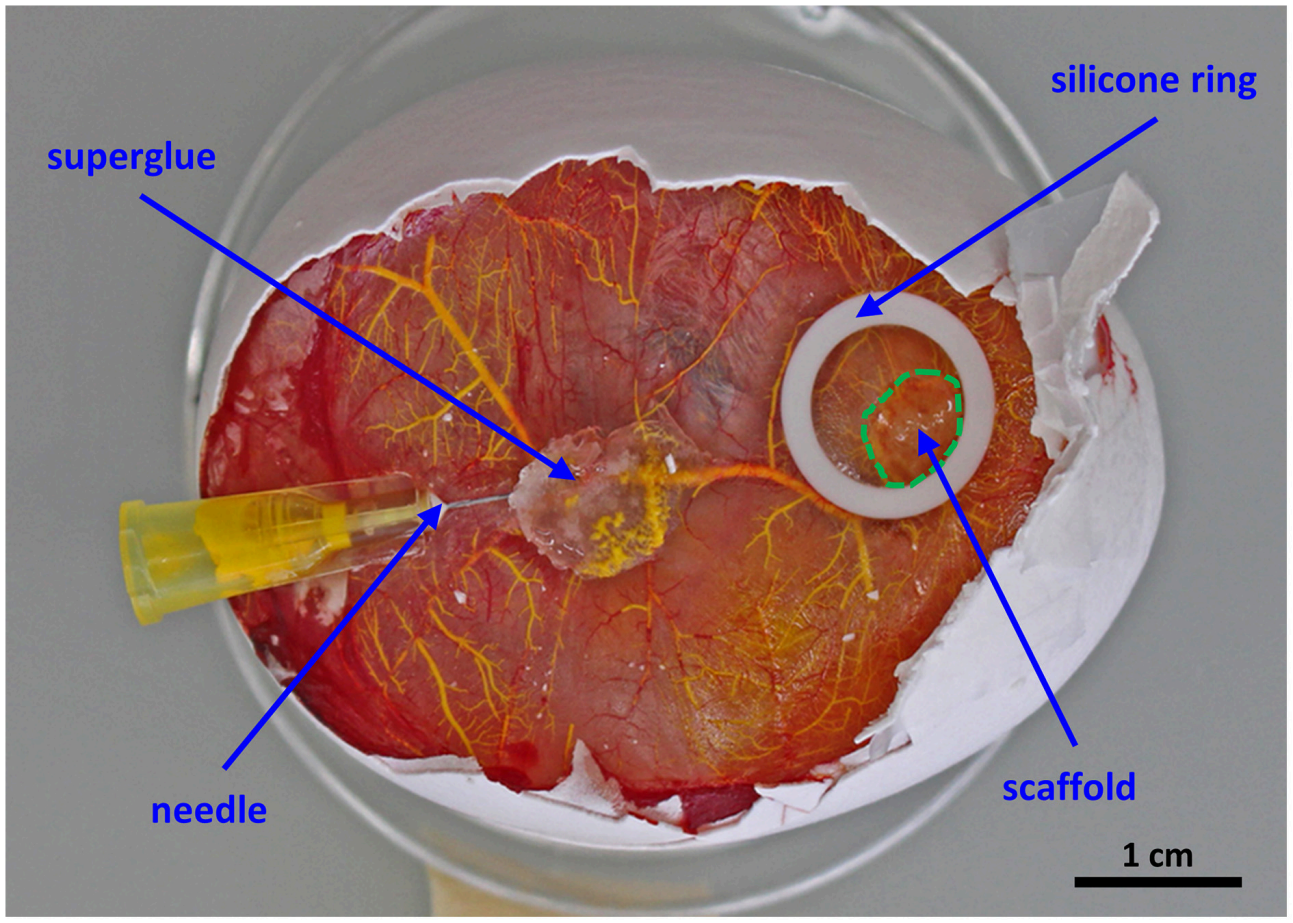
**Setup for the perfusion of the chorioallantoic membrane vasculature**. The scaffold is placed in the middle of a silicone ring and cultured for 7 days *in ovo*. For proper perfusion a 30G ½” needle is fixed with a drop of superglue before MicroFil® (yellow) injection.

- On ED 14, the chicken embryos are perfused with a mix of the MicroFil® components. Dilute the silicone rubber injection compound (yellow) 10-fold in MV-Diluent and add 10% (by weight) MV-Curing Agent right before use. Working time is at least 20 min and starts with the addition of curing agent.
- Fill the MicroFil® mix into a 5 mL syringe and attach a three-way valve (Discofix® C) with a 10 cm tube for more flexibility between the 30G ½” needle and the syringe.
- Using a stereoscope, fix a branch of the vitelline vasculature matching the diameter of the needle with blunt end tweezers and carefully insert the needle into the vessel. Apply a small drop of superglue where the needle enters the vasculature (Figure 1).
- Inject the MicroFil® mix carefully into the vasculature until the pressure increases.
- If filling of the vasculature is not sufficient, inject MicroFil® a second time into a non-perfused vessel. Remove the tube from the needle, which remains in the CAM, and attach a fresh needle to the tube. Repeat steps **c** and **d**.
- Place the perfused chicken embryo overnight at 4°C for complete curing of the MicroFil®.
- On the next day, remove the perfused scaffold samples from the CAM using scissors, wash in PBS, fix in 4% paraformaldehyde, wash in PBS, and place in 70% ethanol until microCT imaging.
- Perform microCT scanning at an isotropic resolution of 20 μm, an energy level of 70 kVp, an intensity of 114 μA, and 300 ms integration time.

According to Swiss animal care guidelines experiments performed in chicken embryos until ED 14 do not need ethical approval (TSchV, Art. 112).

## Results

**Figure 2:**
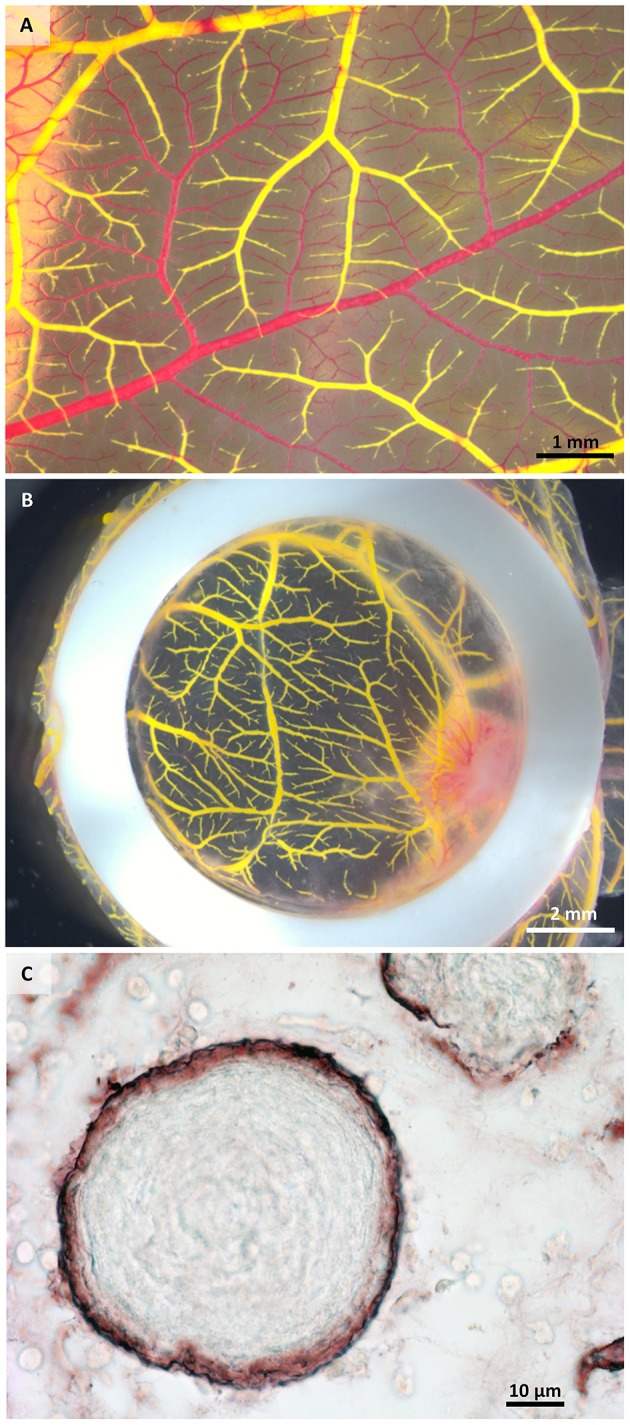
**Macroscopical and microscopical view of perfused samples**. **(A)** Perfused (yellow) and non-perfused (red) vessels after one injection showing incomplete perfusion. **(B)** Exemplary sample after two injections of MicroFil®. **(C)** Histological section of a perfused sample showing vessels (brown) containing MicroFil®.

We have used the CAM assay to study the vascularization of a 3D biomaterial when placed in a highly vascularized environment. The perfusion of the vascular system was performed without pre-perfusion with phosphate buffered saline, as the blood was forced out by the injected MicroFil®. The initial perfusion efficiency with MicroFil® diluted according to the manufacturer's recommendation was improved by using a higher dilution of the silicone rubber injection compound, which required an increased amount of the MV-Curing Agent of 10% instead of 5% (by weight). If the perfusion was not complete after one injection, a second injection site was chosen to fill non-perfused areas (Figures 2A,B). Immunohistological stainings can still be performed on sections of paraffin-embedded samples after perfusion and microCT analysis (Figure 2C).

**Figure 3:**
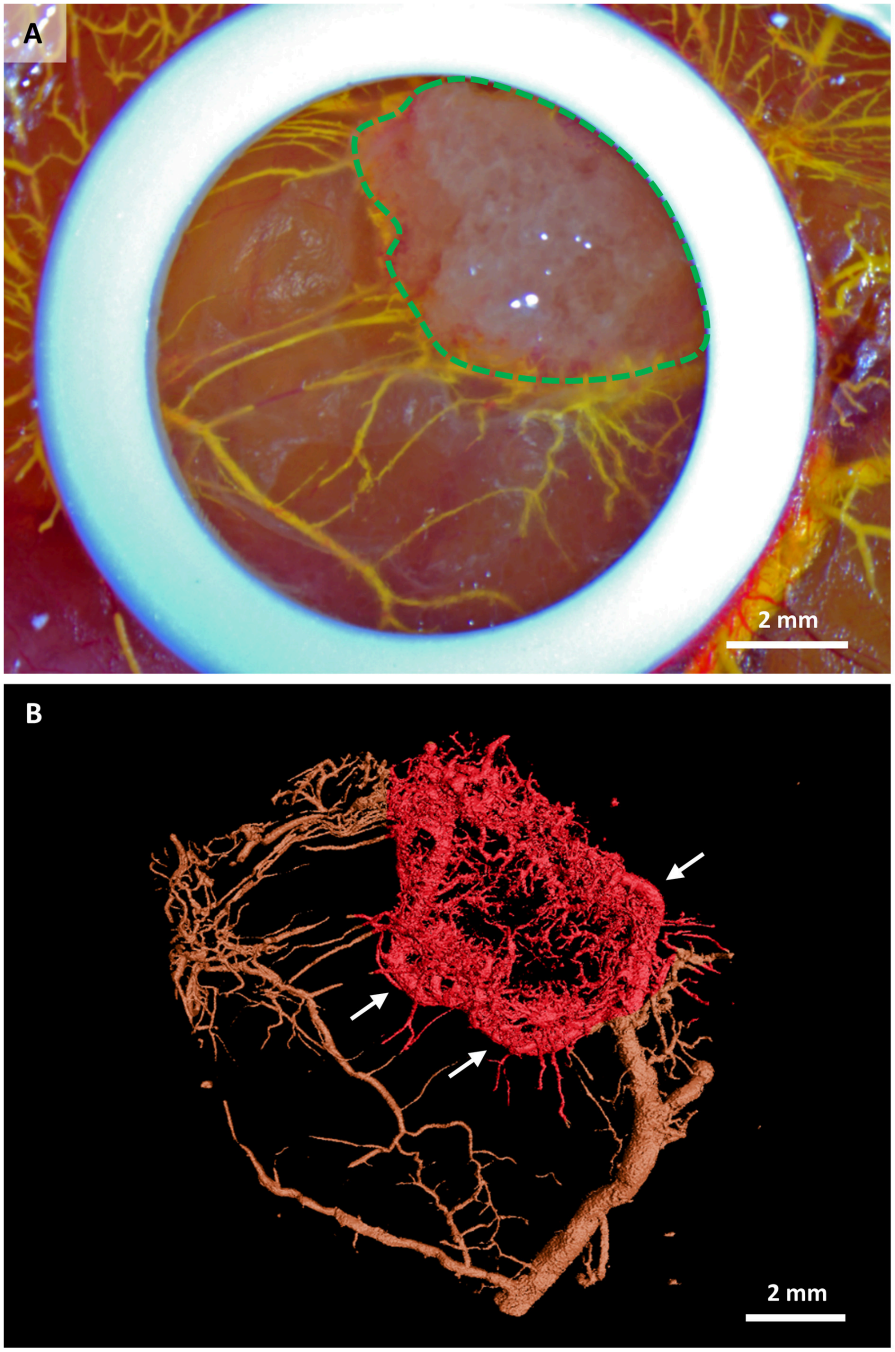
**Stem cell-seeded scaffolds perfused with MicroFil® after their *in ovo* culture. (A)** Macroscopic view of a scaffold (green dashed line) immediately after perfusion with yellow MicroFil®. The silicone ring is seen in white, vasculature in yellow. **(B)** Microcomputed tomography image of the vascular cast within the scaffold (red color). Vasculature outside the scaffold area is marked in brown. New capillaries are sprouting from one large vessel that encircles the scaffold (white arrows).

The microCT scan showed the MicroFil® perfused 3D vascular structure growing into the scaffold (red area in Figure 2B) from the bottom and from the sides within 7 days of *in ovo* culture. While the macroscopical image of the same sample *in ovo* (Figure 3A) provides only the information about the vascularization visible on the surface of the CAM, the microCT image visualizes the whole vascular network. Interestingly, new capillaries are sprouting from one large vessel that encircles the scaffold, (Figure 3B, white arrows). The generated microCT data can be further used for complete morphometric characterization of the vascular network, including the number, length, branching, and diameter of vessels.

## Discussion

The generation of vascularized tissues and organs remains one of the key challenges in regenerative medicine (Novosel et al., 2011; Mitsiadis et al., 2012; Dew et al., 2015). Visualization of angiogenesis within implanted engineered devices aiming at tissue replacement and/or regeneration is pivotal for predicting and understanding the outcome of new regenerative approaches before their application in clinics.

Several techniques have been extensively used to study the vascular architecture of the whole body, as well as the microvasculature of specific organs and tissues in both health and disease. The development of new visualization methods using microcomputed tomography (microCT) improved our knowledge on specific tissue anatomy, physiology, and pathology tremendously. Further, developments using perfusion of tissues and organs before microCT scanning allowed additional analyses at the histological level and generated a plethora of volumetric and quantitative data (Garcia-Sanz et al., 1998). Therefore, the combination of vascular perfusion with microCT imaging and analysis has been applied in many fields, including embryonic vascular development (Anderson-Berry et al., 2005), cancer research (Xuan et al., 2007), cardiac disease models (Sangaralingham et al., 2012), and tissue engineering (Bolland et al., 2008; Stoppato et al., 2013). For example, the combination of MicroFil® perfusion with microCT imaging has been used to study angiogenesis in tissue engineered constructs implanted subcutaneously into immunosuppressed mice. Both natural allografts and osteosynthetic [poly (D,L)-lactic acid; PDLLA] grafts seeded with human bone marrow stromal cells (BMSCs) have been used for this study. The presence of BMSCs was able to increase the number of vessels within the implants (Bolland et al., 2008). Similarly, MicroFil® perfusion and microCT scanning have been performed in VEGF-treated porous polyurethane scaffolds implanted subcutaneously into rats (Schmidt et al., 2009). A more recent study using MicroFil® perfusion and microCT scanning has demonstrated that silk fibroin fibers combined with a PDLLA salt-leached sponge promoted vascularization when implanted subcutaneously into rats (Stoppato et al., 2013).

However, all these previous studies are based on rodents and therefore necessitate extensive animal care-taking, ethical approvals, as well as surgical skills and equipment. Moreover, a minimum of 10 days incubation time is needed in order to analyze implant vasculature. In contrast, the CAM assay is a simple, cost-effective, and highly reproducible method, which does not require ethical approval when performing experiments in chicken embryos until embryonic day 14 (Swiss animal care guidelines, TSchV, Art. 112). Even though the incubation time on the CAM is limited to a period of 7 days, it constitutes an excellent screening method to study the early phase of angiogenesis within implanted scaffolds. Following the “3Rs” principles of replacement, refinement, and reduction of animal use in research, the CAM assay combined with MicroFil® perfusion and microCT analysis as described in this protocol provides a valuable intermediate platform for initial assessments prior to pre-clinical studies in mammals.

This technique could be applied for the direct comparison of scaffolds (ceramics, synthetic polymers, and natural polymers) seeded with stem cell populations of different origins [e.g., adipose-derived stem cells (ASCs), bone marrow stem cells (BMSCs), DPSCs, periodontal ligament stem cells]. For example, it has been shown that BMSCs possess angiogenic potential (Oswald et al., 2004), but little is known about their capability to attract vessels in engineered structures. In contrast, ASCs have been demonstrated to promote the neovascularization when seeded in different scaffolds (De Francesco et al., 2009; Chan et al., 2016; Guo et al., 2016). In conclusion, the present method can constitute a valuable tool for studying in detail cell-mediated vascularization efficiency.

## Author contributions

AW: experimental design, performance of experiments, writing of the manuscript, editing, discussing. DL: experimental design, writing of the manuscript, editing, discussing. TM: experimental design, writing of the manuscript, editing, discussing.

## Funding

This work was supported by the Swiss National Foundation (SNSF) grant 31003A_135633 (TM, AW), by institutional funds from University of Zurich (TM) and by funds from the Second University of Naples (SUN;DL).

### Conflict of interest statement

The authors declare that the research was conducted in the absence of any commercial or financial relationships that could be construed as a potential conflict of interest.
